# Operation for Ruptured Sac in Extra-Uterine Pregnancy

**Published:** 1885-06

**Authors:** 


OPERATION FOR RUPTURED SAC IN EXTRA-UTERINE PREGNANCY.

An editorial appeared in the *Journal of the American Medical Association*, May 23d, 1885, entitled "*Operation for Ruptured Extra-uterine Pregnancy*," which maintains with much of the same positive and unphilosophical dogmatism the position of Wiltshire, Lawson Tait and Knowlsley Thornton with reference to operative interference in all cases of rupture of the cyst in ectopic pregnancy.

An editorial appeared in the *New York Medical Record*, Oct. 25th, 1884, upon the same subject, which contains the following words:

"There is no palliative measure for a ruptured extra-uterine cyst; there is no expectant treatment; and there is no other way known to medicine by which a woman in this condition can be reasonably expected to survive, save by the prompt use of the knife, and there is no reason for thinking that she would die if this be resorted to in time."

It is difficult to conceive of an adequate reason for the indulgence in that *fallacy of generalization* in this connection,—the use of the universal proposition,—unless it be true that "a mixture of lie doth ever add pleasure."

Mr. Lawson Tait's statement, "That the great bulk of such cases (ruptured cyst of ectopic pregnancy) have a fatal termination when left alone," is opposed by an equally positive conviction, expressed by Professor Karl Schrœder: "I, myself, so frequently see cases of tubal pregnancy, in which the diagnosis is positive, pursuing a favorable course, that I consider recovery as the regular termination." (*Karl Schrœder's Lehrbuch d. Geburtshülfe, Bonn. 1884, p. 422.*)

If human opinion is of purely relative value, we are very decidedly of the persuasion that more importance should be attached to the *dictum* of the German observer, as the resultant of greater experience and more extended observation, than to that of the distinguished surgeon of Birmingham. Regard the natural history of the condition:

I. *The sac may rupture, but the egg may remain within and act as a spontaneous tampon.* Such cases, with favorable terminations to the mothers, have been observed by *Wiedersperg* and *Virchow.* (Otto Spiegelberg's Lehrbuch d. Gebertshülfe, Lahr, 1882, p. 290.)

Operative interference under such conditions is plainly contra-indicated.

II. *The cyst may rupture into the ligamentum latum with the formation of a hæmatoma. Schuchardt, and J. Veit* (*Virchow's Arch., Bd., 89, p. 133. Die Eileiterschwangerschaft, von Dr. J. Veit, Stuttgart, 1884*) have observed this termination. The embryo usually dies, and undergoes reductive metamorphosis, while hæmorrhage is checked by the pressure of the folds of the broad ligament. Primary laparotomy in such cases is obviously contra-indicated, unless the hæmorrhage is uncontrollable, or there is reason to believe the embryo will develop in its new location.

IV. *The sac may rupture and the fœtus escape into the peritoneal cavity, with the formation of a retro-uterine hæmatocele. Ollivier, Leclerc, Schrœder, Vignés, Gallard, Karl Braun, Veit, Chiari* and others have observed this mode of termination. Hæmorrhage is a prognostic element of less serious import in these cases than peritonitis. Early operative interference is contra-indicated by the fact that most of these cases recover spontaneously.

V. *The sac may rupture into the peritoneal cavity, and the life of the woman may be threatened by free hæmorrhage, or the resulting peritonitis.* Fatal hæmorrhage even under these circumstances is seldom observed, according to Dr. J. Veit, when rational expectant treatment has been practiced.

In the limited space at our command, we cannot discuss this interesting subject more thoroughly.

Finally, we desire to call the attention of the profession to the concluding paragraph in Dr. J. Veit's monograph, *Die Eileiterschwangerschaft,* as more in consonance with the present state of our knowledge, than the statements of Wiltshire, Lawson Tait, or Knowlsley Thornton.

"*Therefore, I advise, when the diagnosis of uncomplicated tubal pregnancy is made, the extirpation of the sac; in case of the formation of an hæmatocele, as in the death of the fœtus, rest and the expectant plan of treatment; in case of rupture of the cyst into the abdominal cavity, the compression of the abdominal aorta, etc., and only under the most extreme conditions to resort to direct arrest of hæmorrhage by laparotomy.*"

